# Human echinostomiasis: a case report

**DOI:** 10.1186/s13104-018-3133-z

**Published:** 2018-01-11

**Authors:** Ranjit Sah, Shusila Khadka, Rabin Hamal, Sagar Poudyal

**Affiliations:** 1 Department of Microbiology, Tribhuvan University and Teaching Hospital (TUTH), Institute of Medicine, Kathmandu, Nepal; 2 Department of Medicine (Gastroenterology), Tribhuvan University and Teaching Hospital (TUTH), Institute of Medicine, Kathmandu, Nepal

**Keywords:** Echinostomiasis, *Echinostoma* species, Food-borne disease, Abdominal pain, Insufficiently cooked fish and snail, Nepal

## Abstract

**Background:**

Echinostomiasis is a food-borne infection caused by an intestinal trematodes belonging to the family Echinostomatidae. They infect the gastrointestinal tract of humans. Patients are usually asymptomatic. However, with heavy infections, the worms can produce catarrhal inflammation with mild ulceration and the patient may experience abdominal pain, anorexia, nausea, vomiting, diarrhea and weight loss. Infection are associated with common sociocultural practices of eating raw or insufficiently cooked mollusks and fish.

**Case presentation:**

We report a first case of echinostomiasis from Nepal in a 62 years old, hindu male who presented to Tribhuvan University Teaching Hospital, Kathmandu with a complaint of abdominal pain and distension with vomiting on and off for 3–4 months. He had history of consumption of insufficiently cooked fish and snail with alcohol. During endoscopy, an adult flat worm was seen with mild portal hypertensive gastropathy (McCormack’s classification) and erosive duodenopathy. The adult worm was identified as *Echinostoma* species based on its morphology and characteristic ova found on stool routine microscopic examination of the patient. Patient was treated with praziquantel 40 mg/kg (single dose) which is the drug of choice for *Echinostoma* species infection by which he got improved and on follow up stool examination after 2 weeks revealed no ova of *Echinostoma* species.

**Conclusions:**

The patients having history of consumption of insufficiently cooked snail and fish with suggestive clinical features of echinostomiasis should be suspected by physicians and ova of *Echinostoma* species should be searched by trained microscopists. An epidemiological survey is required to know the exact burden of *Echinostoma* species infection in the place where people have habit of eating insufficiently cooked fish and snails, as it can be endemic in that community or geographical area.

## Background

Echinostomiasis is a food-borne parasitic disease caused by an intestinal trematodes belonging to the family Echinostomatidae [1]. It can infect both humans and animals. These intestinal trematodes have a three-host life cycle with aquatic snails as first intermediate hosts in which a sporocyst, two generations of rediae and cercaria develop. Emerged cercariae freely swim and infect the second intermediate hosts, which may be several species of aquatic organisms such as snails, frogs, clams and fishes. Finally, the definite host (human and others animals) become infected after ingestion of the second intermediate host harbouring the encysted metacercariae where an adults worms mature and produce eggs that are released with the host’s feces [2, 3]. They infect the gastrointestinal tract of humans. Patients are usually asymptomatic. However, with heavy infections, the worms can produce catarrhal inflammation with mild ulceration and the patient may experience abdominal pain, anorexia, nausea, vomiting, diarrhea and weight loss [2, 3]. Infection are associated with common sociocultural practices of eating raw or insufficiently cooked mollusks, fish, crustaceans, and amphibians, promiscuous defecation, and use of night soil (human excrement collected from latrines) for fertilization of fish ponds [2, 4].

## Case presentation

### Patient

A 62 year-old, hindu male resident of Gorkha district (28°17′23.92″N, 84°41′23.1″E) at an altitude of 1210 m above sea level and climate zone with an area of 3610 km^2^ and has a population (2001) of 288,134, Province No. 4, Nepal was referred from local government hospital to the Department of Gastro-medicine of Tribhuvan University Teaching Hospital (TUTH) in Kathmandu, Nepal for the evaluation of upper abdominal pain with distension, at the mid of July 2016.

### Anamnesis

The patient had past history of repeated admission for jaundice and abdominal distension probably due to alcoholic liver cirrhosis with decompensation (ascites) in local and regional hospital. This time he had new onset abdominal pain in addition to pre-existing abdominal distension (ascites) with vomiting on and off since 3 months before he got admitted to TUTH. He moreover had a history of consumption of insufficiently cooked fish and snail with alcohol and water cress (aquatic plants) from riverside as the part of his daily meal.

### Clinical picture

In the local government hospital, his abdominal pain was managed with antacids, proton pump inhibitors (PPI), antispasmodic and albendazole but his pain was not relieved. So, he was referred to Tribhuvan University Teaching Hospital where intravenous pantoprazole and hyoscine butylbromide was given which caused only mild relief of pain. Since, his pain was not resolving an ultrasound analysis was performed on 17th July 2016 which showed cirrhotic liver changes with ascites. For further evaluation endoscopy was planned for next day. On 18th July 2016 endoscopy was performed.

A complete blood count of the patient revealed a 72% of neutrophils, 20% of lymphocytes, 7% of eosinophil, 1% of monocytes, and 0% of basophils. Total leucocyte count was 4010/μl of blood and platelet count reached 223,000/μl of blood and hemoglobin was reduced to 10.3 gm/dl.

Patient’s stool sample was collected and processed for routine macroscopic and microscopy examination. On macroscopic examination of stool, it was yellowish–brown with soft consistency.

### Diagnosis

**Fig. 1:**
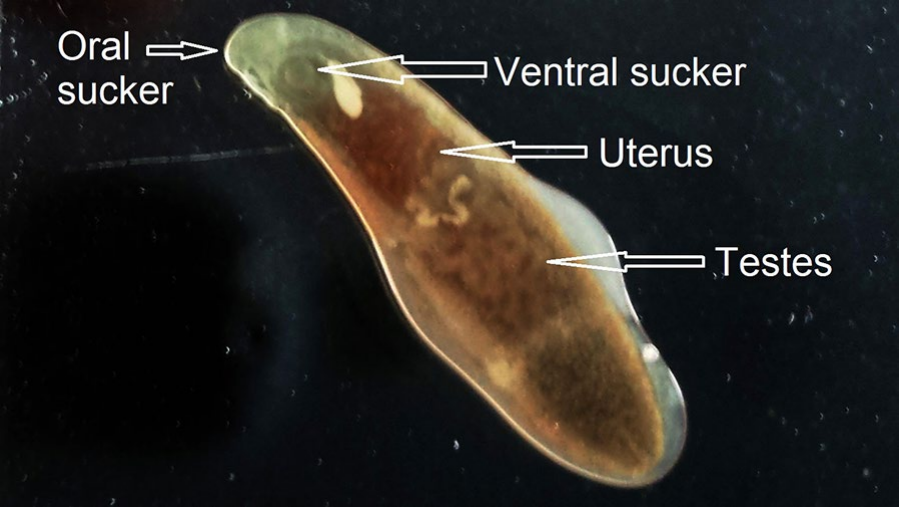
Adult worm of *Echinostoma* species

**Fig. 2:**
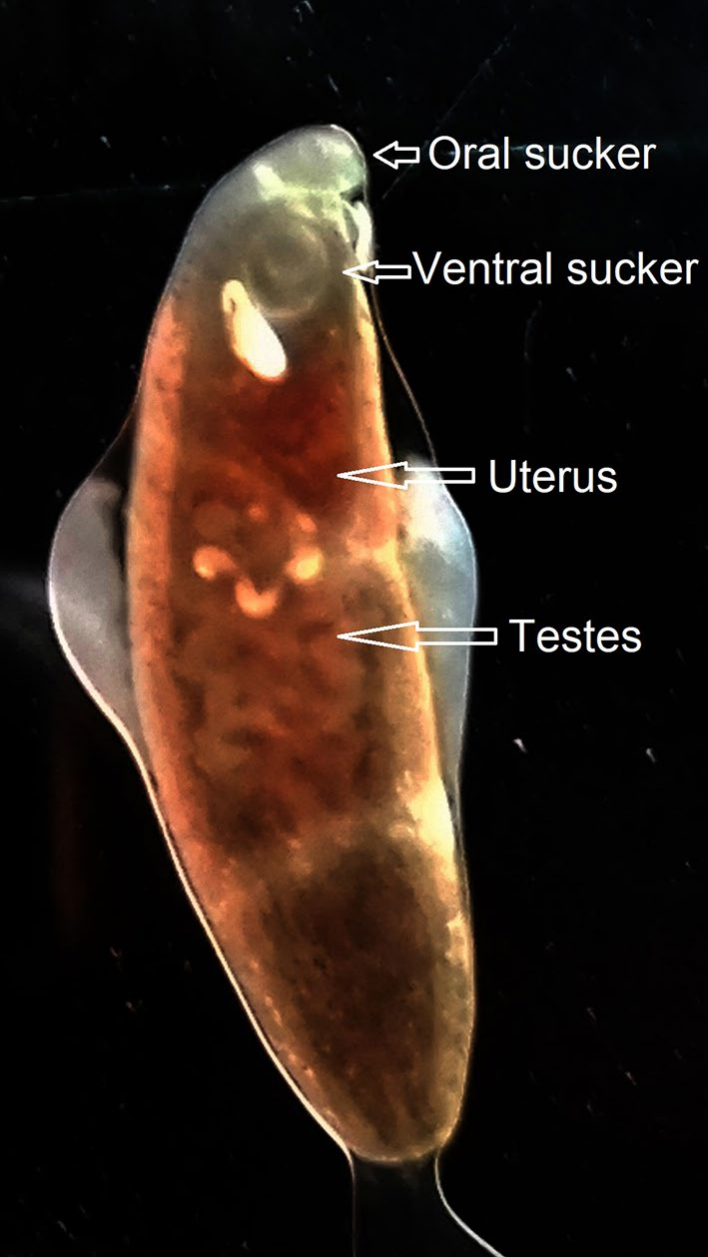
Adult worm of *Echinostoma* species showing oral sucker, ventral sucker, uterus and testes

During endoscopy, an adult flat worm was seen with mild portal hypertensive gastropathy (McCormack’s classification) and erosive duodenopathy. The worm was removed and its morphological characteristics were studied which revealed flat leaf like structure, reddish-gray in color measuring approximately 10 mm in length by 2 mm in width (Figs. 1, 2). Oral sucker, ventral sucker, uterus and testes were clearly observed in the adult worm (Figs. 1, 2) but its head collar with collar spines around the oral sucker were not visible. The adult worm resembled to the *Echinostoma* species, *Clonorchis sinensis* and *Opisthorchis felineus*. Since, neither the facilities for genetic confirmation nor for fixing, staining and mounting of the adult worm was present at our institute, the worm was preserved in 10% formalin and waited until the next morning for the analysis of the stool sample of the patient to reach the possible diagnosis by studying the morphology and characteristics of the ova laid by the worm.

**Fig. 3:**
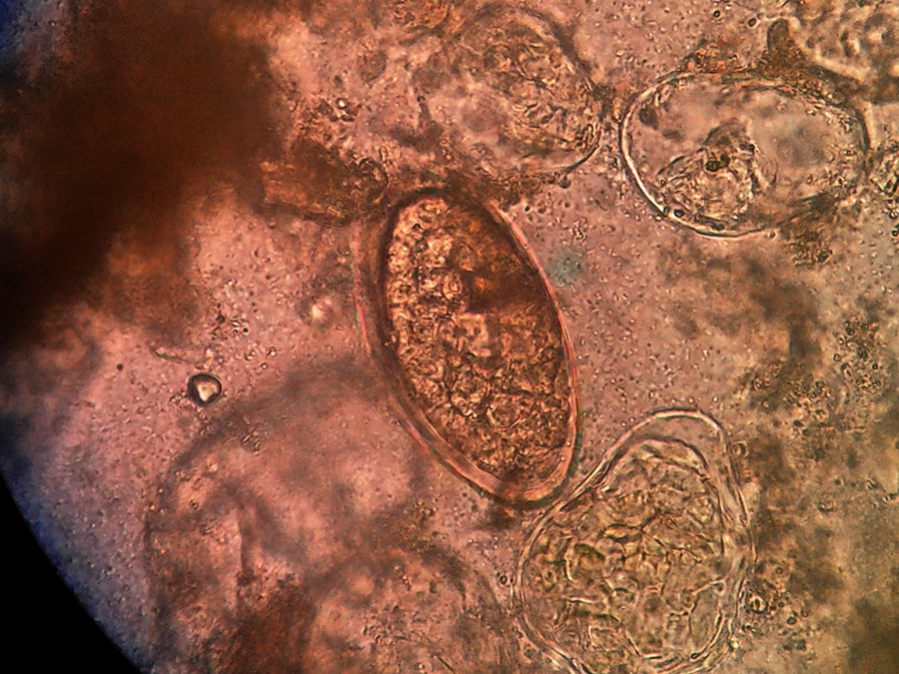
Ova of *Echinostoma* species

**Fig. 4:**
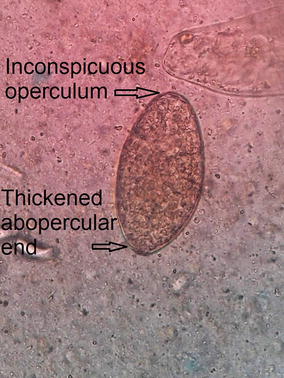
Ova of *Echinostoma* species with somewhat inconspicuous operculum and a thickened, wrinkled abopercular end

**Fig. 5:**
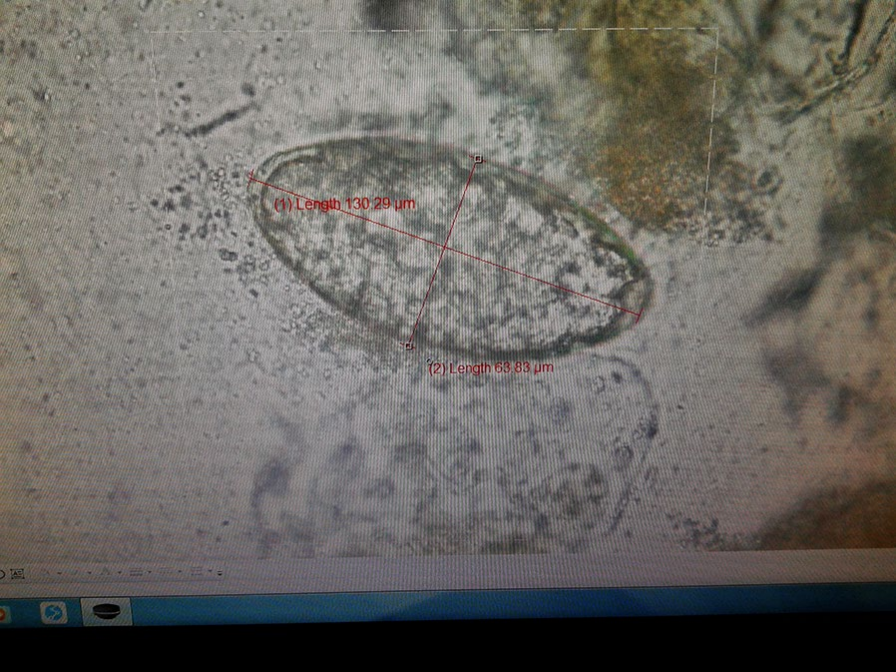
Measurement of the ova (130 μm by 64 μm) using cell sensation software version 1.12 for DP73 camera installed to the Olympus BX53 microscope used for the microscopy

For the microscopic examination of the fecal sample, wet and iodine mount was prepared and examined under light microscope. On wet mount an ellipsoidal, yellow–brown, eggs with somewhat inconspicuous operculum and a thickened, wrinkled abopercular end (Figs. 3, 4) with mean average size of 130 µm by 64 µm (Fig. 5) was observed. The above characteristics of the ova clearly differentiate from the ova of *C. sinensis* and *O. felineus* which have convex operculum that rest on a prominent opercular ‘shoulders’ at the smaller end of the egg and abopercular knob at the opposite end with small size, measuring 27–35 µm by 11–20 µm. The size of the detected ova was measured using cell sensation software version 1.12 for DP73 camera installed to the Olympus BX53 microscope used for the microscopy. On the basis of morphological appearance of adult worm and characteristic feature of the detected ova and its measurement, *Echinostoma* species was identified. The photographic evidence of worm and eggs with the results of their measurement was forwarded to Centers for Disease Control and Prevention (CDC), Atlanta, Georgia, USA and it was confirmed as that of *Echinostoma* species.

**Fig. 6:**
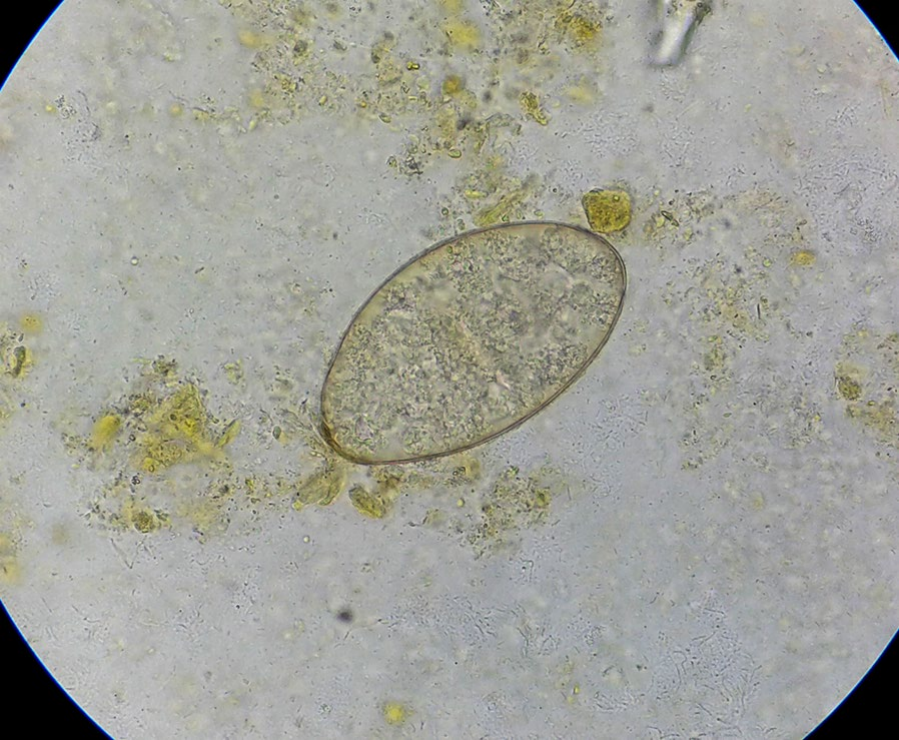
Ova of *Fasciola hepatica* seen in Nepalese patient at Tribhuvan University Teaching Hospital

**Fig. 7:**
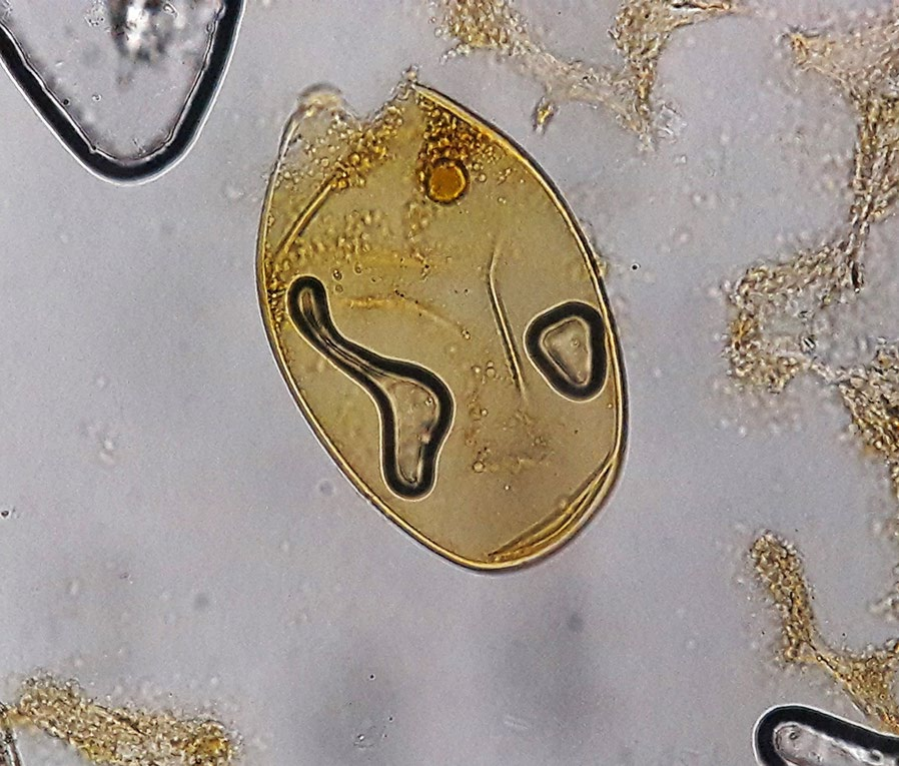
Ova of *Fasciola gigantica* seen in Nepalese patient at Tribhuvan University Teaching Hospital

**Fig. 8:**
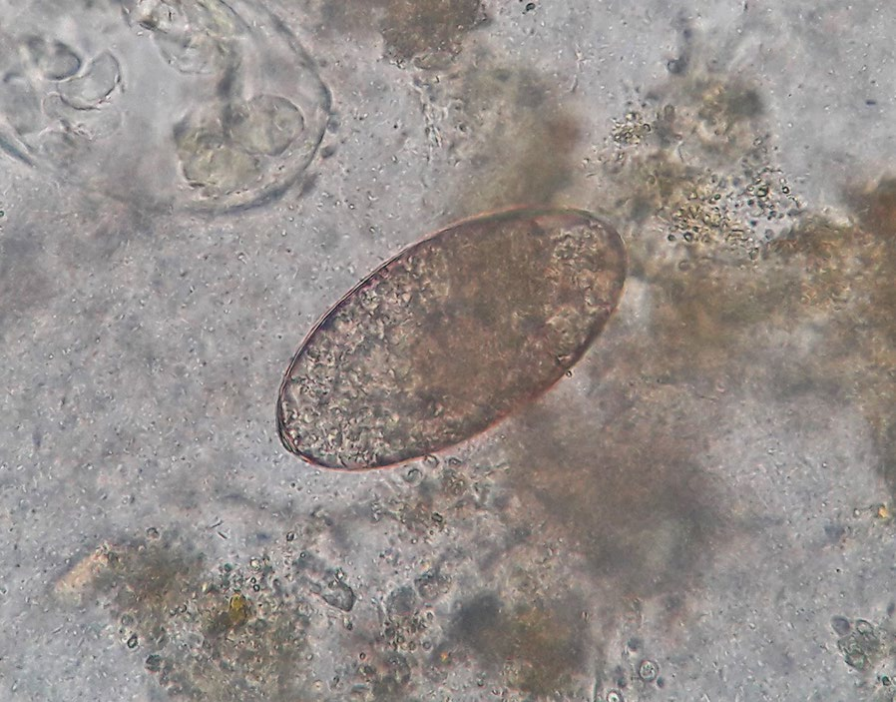
Ova of *Fasciolopsis buski* seen in Nepalese patient at Tribhuvan University Teaching Hospital

**Fig. 9:**
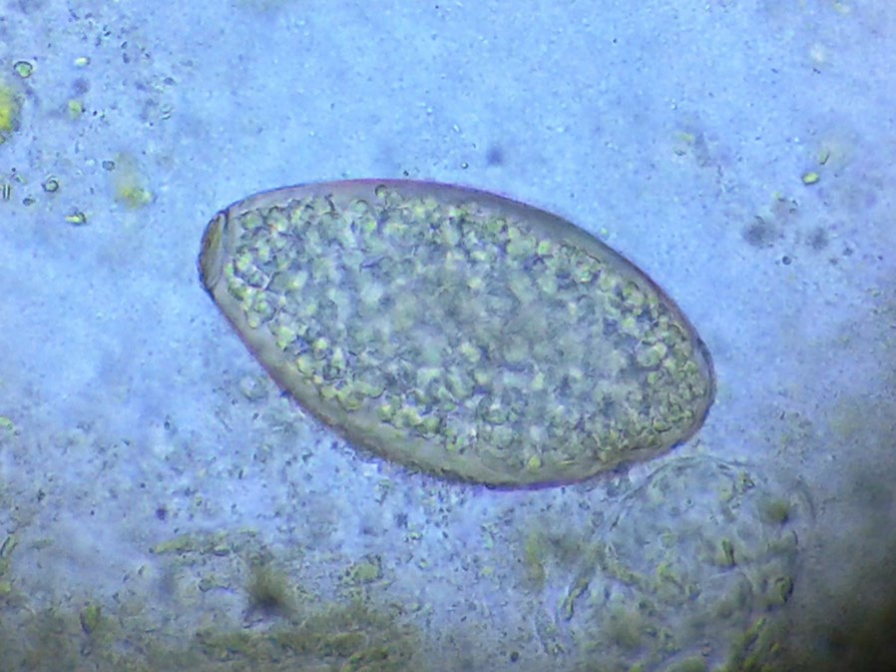
Ova of *Gastrodiscoides hominis* seen in Nepalese patient at Tribhuvan University Teaching Hospital

The ova found in this patient (ova of *Echinostoma* species) resembled closely to the ova of the *Fasciola hepatica*, *Fasciola gigantica*, *Fasciolopsis buski* and *Gastrodiscoides hominis*. To avoid the confusion of co-infection with other trematodes, the ova found in this patient was compared and contrasted with ova of other trematodes available at Tribhuvan University Teaching Hospital, Nepal. The small size, inconspicuous operculum and a thickened, wrinkled abopercular end (Fig. 3) differentiated the ova present in this patient with the closely resembled ova of *F. hepatica* (Fig. 6) which has no thickened or wrinkled abopercular end, *F. gigantica* (Fig. 7) the size of which is larger and without thickened or abopercular end, *Fasciolopsis buski* (Fig. 8) without thickened or wrinkled abopercular end and *G. hominis* (Fig. 9) without thickened or wrinkled abopercular end.

Final diagnosis of *Echinostoma* species was made on the basis of morphological appearance of adult worm and characteristic feature of the detected ova and its measurement.

### Treatment

Patient was treated with praziquantel 40 mg/kg (single dose) which is the drug of choice for *Echinostoma* species infection by which he got improved and on follow up stool examination after 2 weeks revealed no ova of *Echinostoma* species with confirmed resolution of the abdominal pain.

## Discussion and conclusions

To our knowledge, this is the first report of *Echinostoma* species infection in a human in Nepal, although echinostomiasis is regarded as endemic in Southeast Asia. Echinostomiasis is a food-borne, intestinal, zoonotic parasitosis usually infecting birds and mammals and only 23 species are known to cause infections in human [5, 6]. It is distributed worldwide but most frequently seen in South-East Asian countries [5]. The main source of infection is large fresh water snails *Viviparus javanicus* (in the Indonesia) and *Pila luzonica* (in the Philippines) and dogs and rats are animal reservoir hosts [7]. *Echinostoma ilocanum* was first discovered in residents of Manila, Philippines in 1907 [7]. Later, human infection with *E. ilocanum* was reported from Indonesia, China, Thailand and India [8]. *E. malayanum* was first reported by Leiper in labourers of Indian origin in Malaysia [9]. In case of heavy worm load in *E. malayanum*, mortality due to anaemia, malnutrition and intestinal perforation have been reported [7]. A study in Thailand by Peng et al. showed that, among the total (1364) stool sample, 18% (245) were positive for one or more parasites and *Echinostoma* species accounted to 0.1% [10]. According to Lee et al. from Seoul Paik Hospital revealed egg positivity rate of *Echinostoma* species as 0.03% (12) out of total 53,552 fecal specimens [11]. An epidemiological survey by Ryang from Korea in 473 junior high school students and 169 inhabitants revealed 3 (0.5%) positive cases of echinostomiasis [12].

In our neighboring country India, one case report of *E. malayanum* was done in a tribal community near Calcutta in 1993 and first reported case of another species *E. ilocanum* was done in 1998 in New Delhi in a girl residing in Bihar [5]. A total of nine species have been reported infecting human in China with the prevalence of 3.2% where *E. fujianensis* is the most common species [3].

In present case, the parasite was identified as *Echinostoma* species whose adult worm resembles to the adult worm of *Clonorchis sinensis* and *Opisthorchis felineus* but differ in egg morphology and size. The ova of *Echinostoma* species had inconspicuous operculum with a thickened, wrinkled abopercular end with measurement 130 µm by 64 µm while the ova of *C. sinensis* and *Opisthorchis* species are comparatively small and have the prominent operculum with knob at the abopercular end [13].

The adult worm of *Echinostoma* species differ from that of the *C. sinensis* and *Opisthorchis* species in that, the former have the presence of a head collar with collar spines around the oral sucker and the spine of the cephalic collar may be arranged in one or two circle [1].

In the present case, collar spine could not be demonstrated due to lack of facilities of staining and electron microscopy. The worm can be fixed into 70% alcohol and stained with semichon’s acetocarmine [14]. In the places where staining facilities and genetic confirmation is not possible, morphology of both the adult worm and ova should be studied to reach the diagnosis, as in this case.

The eggs of *Echinostoma* species resemble the eggs of *F. hepatica*, *F. gigantica*, *F. buski* and *G. hominis* in shape, color, content and even overlap in their measurement. The presence of inconspicuous thin operculum and a thickened, wrinkled abopercular end of ova of *Echinostoma* species facilitates the differentiation from the ova of other trematodes but the definite differentiation can be made by studying and demonstrating the adult form of *F. hepatica* [15, 16], *F. gigantica* [15, 16], *F. buski* [17] and *G. hominis* [18] which can be easily differentiated from the *Echinostoma* species [5].

Echinostomiasis is not reported from Nepal, although many cases has been reported in our neighboring countries China and India. Rivers originating in China flows through Nepal and reaches India harbouring the intermediate host for *Echinostoma* species.

*Echinostoma* species have rarely been identified in humans probably because of its difficulty in diagnosis by fecal examination as the eggs produced per worm is low in comparison to other helminthic parasites and the eggs are unfamiliar to laboratory personnel and clinicians. So training of the microscopists is a must to detect the eggs of *Echinostoma* species. The patients having history of consumption of insufficiently cooked snail and fish with suggestive clinical features of echinostomiasis should be suspected by physicians and ova of *Echinostoma* species should be searched by trained microscopists. An epidemiological survey is required to know the prevalence of infection, as it can be endemic in that community or geographical area.

## Abbreviations

*E. ilocanum*: *Echinostoma ilocanum*
*E. malayanum*: *Echinostoma malayanum*
*E. fujianensis*: *Echinostoma fujianensis*
*C. sinensis*: *Clonorchis sinensis*
*O. felineus*: *Opisthorchis felineus*
*F. hepatica*: *Fasciola hepatica*
*F. gigantica*: *Fasciola gigantica*
*F. buski*: *Fasciolopsis buski*
*G. hominis*: *Gastrodiscoides hominis*
TUTH: Tribhuvan University Teaching Hospital
CDC: Centers for Disease Control and Prevention
USA: United States of America
µl: microliter
µm: micrometer
mm: millimeter
g/dl: gram per deciliter

## References

[1] Toledo R, Esteban JG (2016). An update on human echinostomiasis. Trans R Soc Trop Med Hyg.

[2] Garcia LS (2007). Diagnostic medical parasitology.

[3] Toledo R, Muñoz-Antoli C, Esteban JG (2014). Intestinal trematode infections. Adv Exp Med Biol.

[4] Graczyk TK, Fried B (1998). Echinostomiasis: a common but forgotten food-borne disease. Am J Trop Med Hyg.

[5] Grover M, Dutta R, Kumar R, Aneja S, Mehta G (1998). *Echinostoma ilocanum* infection. Indian Pediatr.

[6] Waikagul J (1991). Intestinal fluke infections in Southeast Asia. Southeast Asian J Trop Med Public Health.

[7] Chai JY, Fried B, Toledo R (2009). Echinostomes in humans. The biology of echinostomes.

[8] Sohn WM, Kim HJ, Yong TS, Eom KS, Jeong HG, Kim JK (2011). Echinostoma ilocanum Infection in Oddar Meanchey Province, Cambodia. Korean J Parasitol.

[9] Leiper RT (1911). A new echinostome parasite in man. J Lond School Trop Med..

[10] Peng HW, Chao HL, Fan PC (1993). Imported *Opisthorchis viverrini* and parasite infections from Thai labourers in Taiwan. J Helminthol.

[11] Lee SK, Shin BM, Chung NS, Chai JY, Lee SH (1994). Second report on intestinal parasites among the patients of Seoul Paik Hospital (1984–1992). Korean J Parasitol.

[12] Ryang YS (1990). Studies on *Echinostoma* spp. in Chungju Reservoir and upper streams of Namhan River. Korean J Parasitol.

[13] Centers for disease control and prevention. DPDx - Laboratory identification of parasitic diseases of public health concern. Stool specimens - intestinal parasites: comparative morphology tables; 2016. https://www.cdc.gov/dpdx/diagnosticprocedures/stool/morphcomp.html. Accessed 30 Aug 2017.

[14] Cho Chang-Min, Tak Won-Young, Kweon Young-Oh, Kim Sung-Kook, Choi Yong-Hwan, Kong Hyun-Hee (2003). A human case of *Echinostoma hortense* (Trematoda: Echinostomatidae) infection diagnosed by gastroduodenal endoscopy in Korea. Korean J Parasitol.

[15] Sah R, Khadka S, Khadka M, Gurubacharya D, Sherchand JB, Parajuli K (2017). Human fascioliasis by *Fasciola hepatica*: the first case report in Nepal. BMC Res Notes..

[16] Periago MV, Valero MA, Panova M, Mas-Coma S (2006). Phenotypic comparison of allopatric populations of *Fasciola hepatica* and *Fasciola gigantica* from European and African bovines using a computer image analysis system (CIAS). Parasitol Res.

[17] Mohanty I, Narasimham MV, Sahu S, Panda P, Parida B (2012). Live *Fasciolopsis buski* vomited out by a boy. Ann Trop Med Public Health.

[18] Mas-Coma S, Bargues MD, Valero MA (2005). *Fascioliasis* and other plant-borne trematode zoonoses. Int J Parasitol.

